# The Impact of Oxidative Stress on the Bone System in Response to the Space Special Environment

**DOI:** 10.3390/ijms18102132

**Published:** 2017-10-12

**Authors:** Ye Tian, Xiaoli Ma, Chaofei Yang, Peihong Su, Chong Yin, Ai-Rong Qian

**Affiliations:** Key Laboratory for Space Bioscience and Biotechnology, Bone Metabolism Lab, School of Life Sciences, Northwestern Polytechnical University, Xi’an 710072, China; tianye@nwpu.edu.cn (Y.T.); xiaoli225@mail.nwpu.edu.cn (X.M.); chaofei-yang2015@mail.nwpu.edu.cn (C.Y.); suph@mail.nwpu.edu.cn (P.S.); yinchong42@mail.nwpu.edu.cn (C.Y.)

**Keywords:** oxidative stress, bone loss, microgravity, radiation, countermeasure

## Abstract

The space special environment mainly includes microgravity, radiation, vacuum and extreme temperature, which seriously threatens an astronaut’s health. Bone loss is one of the most significant alterations in mammalians after long-duration habitation in space. In this review, we summarize the crucial roles of major factors—namely radiation and microgravity—in space in oxidative stress generation in living organisms, and the inhibitory effect of oxidative stress on bone formation. We discussed the possible mechanisms of oxidative stress-induced skeletal involution, and listed some countermeasures that have therapeutic potentials for bone loss via oxidative stress antagonism. Future research for better understanding the oxidative stress caused by space environment and the development of countermeasures against oxidative damage accordingly may facilitate human beings to live more safely in space and explore deeper into the universe.

## 1. Introduction

After the Moon landing in 1969, humankind never stop exploring the universe. For example, Shenzhou programs and the International Space Station (ISS) that are orbiting around the Earth recruit crew members continuously [1,2], and several research programs have been launched towards the Moon [3,4]. Even the Mars journey has been gradually industrialized [5,6]. We are standing in the space age now. Although space traveling sounds fascinating, it can cause dramatic changes of the human body, especially long-term spaceflights. More and more evidence proves that the space environment negatively affects human physiological functions with the extension of space stays. Gravitational unloading due to microgravity and cosmetic rays are conditions experienced by astronauts during space flight. The medical examinations conducted before, during and after spaceflight have revealed several health issues for space travelers, e.g., cardiovascular dysfunction, disruption in nervous system, and reduced immune function [7,8,9,10,11]. Bone loss induced by microgravity is also a well-documented alteration in astronauts [12,13,14]. It happens especially on weight-bearing bones and needs a very long duration to recover after returning to earth [15]. In the absence of countermeasures, this change can impact the performance and safety of crew members severely during extravehicular activities, and putting them at high risk of fracture [16]. Bone loss is one of the major obstacles to space exploration for human beings now.

Nowadays, researchers make great efforts to catch the mechanisms hidden behind the physiological alterations of bone during spaceflight and to develop countermeasures accordingly. Russian investigators found reductions in some blood antioxidants and increased lipid peroxidation in human after long-term space flight [17,18]. Urinary excretion of 8-iso-prostaglandin F2α and 8-oxo-7,8 dihydro-2 deoxyguanosine, which are markers of oxidative damage to lipids and DNA respectively, increased during and after long-duration space flight (90 to 180 days) [19]. It means that the balance between oxidant production and antioxidant defenses has been disturbed, and the excessive oxidants may attack DNA and membrane lipids resulting in oxidative damage. The pro-oxidative conditions caused by space environment may contribute to the bone alterations after long space habitation.

In this review, we summarized the oxidative effect to bone caused by microgravity and radiation, and expounded the relationship between oxidative stress and bone formation. The possible mechanisms will be discussed as well. Some prevention countermeasures of bone loss against oxidative injuries will be included too. This manuscript will help to capture the latest research progresses and inspire the possible direction of future studies.

## 2. Effects of Oxidative Stress on Bone Formation

The redox balance in the human body is maintained delicately, with the balance slightly inclined to oxidants [20]. Reactive oxygen species (ROS) are generated as normal by-products of aerobic metabolism, usually by leakage from the electron transport chain during oxidative phosphorylation in mitochondria [20,21]. The major forms of ROS include the superoxide anions (O_2_^−^, hydrogen peroxide (H_2_O_2_) and free radicals such as hydroxyl radicals (OH·). ROS at lower concentrations serve as signaling molecules to activate specific physiologic pathways that control several life processes [22,23]. Meanwhile, elevated levels of ROS can damage proteins, lipids, and DNA, eventually trigger oxidative stress and leading to cell death [24,25]. Oxidative damage to bio-macromolecule has been proved in the etiology of a wide variety of acute and chronic diseases, including osteoporosis [26].

**Scheme 1:**
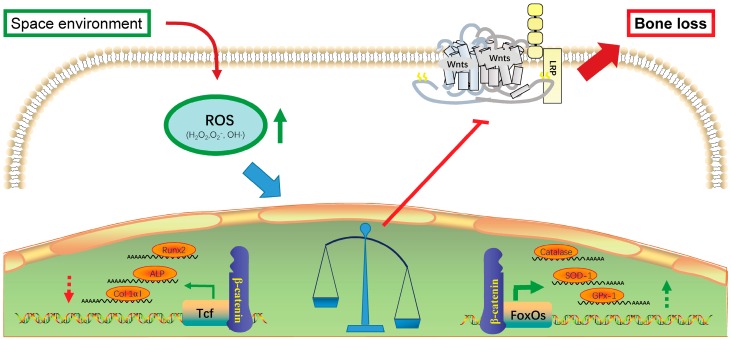
The scheme of possible mechanism of space environment-induced bone loss: the increment of ROS caused by Space environment antagonizes the skeletal effects of Wnt/β-catenin/Tcf by diverting β-catenin from Tcf—to FoxOs-mediated transcription. LRP: LDL receptor-related proteins.

It is reported that the increased level of ROS had opposite effects on osteoblast and osteoclast cells. ROS inhibits osteoblast function. It is believed that the increased level of ROS in osteoblast is one critical element of the pathophysiology of bone loss [27,28,29,30]. Almeida et al. reported that ROS inhibited osteoblast differentiation and promoted apoptosis [31,32,33]. ROS achieve this function by activating a small family of transcription factors known as Forkhead box O (FoxO), which contains four members: FoxO1, FoxO3a, FoxO4, and FoxO6 [34]. FoxOs defense ROS by up-regulating free radical scavenging enzymes such as Catalase, manganese superoxide dismutase (Mn-SOD), and glutathione peroxidase-1 (GPx-1) [35]. Importantly, FoxO-mediated transcription requires the binding of β-catenin that is also essential companion for T-cell factor (Tcf) family of transcription factors [36,37]. Without Tcf transcriptional activities, the downstream effects of the Wnt/β-catenin pathway cannot be conducted [37,38]. Thus, by competitive binding to β-catenin, FoxOs antagonizes Wnt/Tcf-mediated transcription after being activated by ROS (Scheme 1) [39]. Regarding the importance of Wnt/β-catenin/Tcf to bone formation, the attenuation of this pathway will inevitably lead to decreased osteogenesis [40]. Several researchers indicated that conditional deletion of FoxOs (FoxO1, FoxO3, FoxO4) in mice osteoblast resulted in a decrease in the number of osteoblasts, the rate of bone formation and bone mass but an increase of osteoblast apoptosis and oxidative stress in bone [41,42].

On the contrary, ROS play crucial roles in osteoclast differentiation and function. By increasing receptor activator of nuclear factor-kappa B ligand (RANKL) production and activating ERK/NF-κB/TNF/interleukin 6, ROS inhibit osteoclast apoptosis and promote osteoclastogenesis [29]. In addition, it is reported that RANKL could suppress the transcriptional activity of FoxOs, loss FoxOs’ transcription factor function promoted osteoclast differentiation and survival, because intracellular H_2_O_2_ accumulation is pivotal for osteoclastogenesis and bone resorption [43]. Therefore, FoxOs are crucial regulators of both osteoblast and osteoclast physiology, and direct mechanistic links between oxidative stress and skeletal involution.

Another oxidative stress-related pathway includes Nrf2/HO-1, which also can adjust cellular ROS via a switch on gene transcription of several antioxidative enzymes such as SOD, Catalase, GPx, etc. [44]. Mitochondrial dynamics [45], endoplasmic reticulum stress pathway [46], and autophagy [47] are also participated in the bone loss induced by oxidative stress.

## 3. Microgravity Increases Oxidative Stress in Bone System

Microgravity conditions in space cause an imbalance between bone formation and resorption [48,49,50,51]. The average rate of aBMD loss is 1–1.5% per month evaluated by dual-energy X-ray absorptionmetry (DXA) scans from preflight and postflight [52]. Bone loss under microgravity conditions is relevant to oxidative injury. Microgravity is considered to increase free radical formation and causes oxidative stress [53,54,55,56]. Findings either from the real spaceflight missions or ground-based models (head-down bed rest model and hind-limb unloading rodents) all demonstrated elevated oxidative damage markers and attenuated total antioxidant capacity [53,57,58]. Hind-limb unloading (HLU) rodents, rotary wall vessel bioreactor (RWVB) and Random Positioning Machines (RPMs) are commonly used microgravity models in vivo and in vitro. Xin et al. and Sun et al. both observed that malondialdehyde levels (oxidant marker) were raised but total sulfhydryl content (anti-oxidant marker) descended in femurs of HLU Sprague-Dawley (SD) rats [59,60]. MC3T3-E1 cells that were exposed to RWVB had higher cellular ROS levels but lower differential abilities [59,60]. On the contrary, RWVB treatment-induced ROS generation facilitated osteoclastogenesis of RAW264.7 cells [59,60]. Their findings illustrated that the generation of ROS increased in response to microgravity. The excessive ROS destroyed normal function of osteoblasts but enhanced the osteoclasts’ capabilities, which lead to insufficient bone formation and massive bone absorption.

It is believed that the oxidative damage caused by the space environment is related to insufficient nutrition intake and disturbed iron metabolism as well [54,55]. By analyzing blood and urine samples from 23 crew members who participated in missions lasting 50 to 247 days on the ISS, Zwart et al. found serum ferritin was positively correlated with 8-hydroxy-2′-deoxyguanosine (*r* = 0.53, *p* < 0.001) and prostaglandin F2α (*r* = 0.26, *p* < 0.001), which are oxidative damage makers [55]. In addition, they revealed that greater amount of ferritin during flight is accompanied by greater loss in bone mineral density in the total hip (*p* = 0.031), trochanter (*p* = 0.006), hip neck (*p* = 0.044), and (*p* = 0.049) after flight [55]. Their research inspired us that microgravity-induced bone loss may be associated with oxidative stress caused by increased iron store.

Besides iron metabolism, the downregulation of anti-oxidative enzymes like Mn-SOD are also key reasons for oxidative stress-induced bone loss in response to microgravity [61,62,63]. The deficiency of anti-oxidative enzymes can cause distinct weakness in bone and bone fragility [64], and dysfunctional oxidative defense system will exacerbate bone loss via suppressed osteoblastic abilities during mechanical unloading [65].

In brief, microgravity affects oxidative status of bone in many aspects. Mechanical unloading-induced bone loss is closely associated with increased ROS level in different types of bone cell in response to microgravity. Through disturbing oxidative-antioxidative defense systems, microgravity breaks the equilibrium between bone formation and bone absorption leading to skeletal fragility.

## 4. Radiation Induces Oxidative Stress in Bone System

In addition to microgravity, cosmic radiation is another predominant feature of the space environment, and it is a strong incentive to oxidative stress [66,67,68,69]. To date, direct research in spaceflight about the connection between oxidative stress caused by radiation and bone involution is rare. However, some ground-based study suggested the inhibitory effect of radiation to bone formation. Irradiation suppressed bone-like nodule formation, alkaline phosphatase (ALP) activity and expression of osteoblast markers in MC3T3-E1 cells [70]. Meanwhile, the depletion of antioxidant defense enzymes and accumulation of cellular ROS were observed [70]. A similar phenomenon was also exhibited in bone marrow-derived skeletal cell progenitors after a single dose (1–5 Gy) irradiation (137Cs Gy/min) exposure [71]. In an HLU mouse model, total body gamma irradiation (1 or 2 Gy of 137Cs) to C57BL/6 mice decreased cancellous bone volume fractions in the proximal tibiae and lumbar vertebrae significantly, but increased osteoclast surface 47% in the tibiae [72]. Irradiation to total body also stimulated generation of ROS in marrow cells and promoted cell apoptosis [72]. These results inferred that irradiation may cause oxidative stress and inhibit the osteoblasts’ growth and differentiation, but encourage bone absorption. Thus, cosmic radiation may affect critical bone cell functions by stimulating production of ROS, and its suppressive effect to osteoblast involved oxidative stress-mediated activation of Nrf2/HO-1 pathway [70].

## 5. Countermeasures against Bone Loss Caused by Oxidative Stress in Spaceflight

The development of effective countermeasures against oxidative damage in bone during long-term spaceflight is essential. Expanded investments in ground-based or in-flight studies revealed some approaches for antagonism of oxidative stress triggered by microgravity and cosmic radiation.

Adequate intake of antioxidant vitamins (e.g., vitamins C and E and carotenoids) can reduce oxidative damages in bones [73,74]. Some research indicated drinking of hydrogen water could relieve microgravity-induced reduction of bone mineral density and augmentation of malondialdehyde in bone tissue [60]. In addition, consuming a diet that provides other naturally occurring antioxidants, such as carotenoids and flavonoids is also effective to reverse microgravity-induced skeletal involution. For example, curcumin, a phenolic natural product isolated from the rhizome of Curcuma Longa (turmeric), could attenuate HLU-induced bone loss by suppressing oxidative stress [59].

Some natural products have exhibited skeletal benefits against oxidative stress. Tanshinol, extracted from Salvia miltiorrhiza Bunge, rescued the decrease of osteoblastic differentiation via down-regulation of FoxO3a signaling and upregulation of Wnt signal under oxidative stress [75]. Some extracts from teas also have osteogenic benefits against oxidative stress [76,77]. Other antioxidants, e.g., α-lipoic acid and N-acetyl cysteine, could restore the changes induced by oxidative stress in bone as well [70,72]. Although these data are not from ground-based models or in-flight studies, they can still enlighten the development of countermeasures against bone loss induced by oxidative stress during space flight.

Humans are embarking on the adjustment of diet in long-duration space flight. It seems that intake of rich antioxidants will prevent oxidative damage caused by space environment. With the progress of research, certain medical approaches will be created and we will conquer oxidative stress-induced bone loss eventually.

## 6. Conclusions and Perspectives

Space is a stressful environment. Microgravity and cosmic rays are main adverse factors challenging the survival of organisms. The oxidative stress triggered by spaceflight causes a variety of damages to the human body including skeletal involution. In this review, we summarized the stimulation of oxidative stress by radiation and microgravity in space, and its inhibitory effect on bone formation. We discussed the possible mechanisms of oxidative injury induced by space environment to the bone system. Presently, it is mainly believed that the attributed factors include inadequate nutrition intake, increased iron store, and an impaired oxidative defense system. Some countermeasures that have therapeutic potentials for bone loss via oxidative stress antagonism are also mentioned in this manuscript.

Although some progress has been made, the mechanisms of oxidative injuries induced by space habitation are not fully understood. For example, how is gravity sensed and transduced in the bone system and how does it cause the elevation of ROS correspondingly? How do ROS cause bone loss under the special space environment? Further steps need to be taken to thoroughly clarify the whole predisposing process of oxidative stress in space flight and mechanotransduction of gravity, and to develop countermeasures accordingly. It is understandable that resources are limited for spaceflight itself, because the crew time and sample return are restricted and the subject pools are small. Therefore, some ground-based models can be used as vital experimental platforms that allow researchers to examine the effects of the special space environment on bone system. To date, researchers suggest the potential application of antioxidants as a useful dietary source in astronauts’ lifestyles. Perhaps it is one solution for oxidative injury during long-term space habitation. The development of countermeasures against oxidative damage will facilitate human beings residing longer in space and truly entering the space era.

## Abbreviations

ROS	Reactive Oxygen Species
HLU	Hind-limb Unloadings
ISS	International Space Station
FoxO	Forkhead box O
Mn-SOD	Manganese superoxide dismutase
GPx-1	Glutathione peroxidase-1
Tcf	T-cell factor
LRP	LDL receptor-related proteins
aBMD	Areal bone mineral density
DXA	Dual-energy X-ray absorptionmetry
RWVB	Rotary wall vessel bioreactor
SD	Sprague-Dawley
RANKL	Receptor activator of nuclear factor-kappa B ligand

## References

[1] Williams D.R., Turnock M. (2011). Human space exploration the next fifty years. Mcgill. J. Med..

[2] Zhao L., Gao Y., Mi D., Sun Y. (2016). Mining potential biomarkers associated with space flight in Caenorhabditis elegans experienced Shenzhou-8 mission with multiple feature selection techniques. Mutat. Res..

[3] Fu Y., Li L., Xie B., Dong C., Wang M., Jia B., Shao L., Dong Y., Deng S., Liu H. (2016). How to Establish a Bioregenerative Life Support System for Long-Term Crewed Missions to the Moon or Mars. Astrobiology.

[4] Byloos B., Coninx I., Van Hoey O., Cockell C., Nicholson N., Ilyin V., Van Houdt R., Boon N., Leys N. (2017). The Impact of Space Flight on Survival and Interaction of Cupriavidus metallidurans CH34 with Basalt, a Volcanic Moon Analog Rock. Front. Microbiol..

[5] Witze A. (2016). NASA rethinks approach to Mars exploration. Nature.

[6] Caceres M. (2005). Creating a space exploration industry. Aerosp. Am..

[7] Demontis G.C., Germani M.M., Caiani E.G., Barravecchia I., Passino C., Angeloni D. (2017). Human Pathophysiological Adaptations to the Space Environment. Front. Physiol..

[8] Otsuka K., Cornelissen G., Furukawa S., Kubo Y., Hayashi M., Shibata K., Mizuno K., Aiba T., Ohshima H., Mukai C. (2016). Long-term exposure to space’s microgravity alters the time structure of heart rate variability of astronauts. Heliyon.

[9] Mao X.W., Nishiyama N.C., Pecaut M.J., Campbell-Beachler M., Gifford P., Haynes K.E., Becronis C., Gridley D.S. (2016). Simulated Microgravity and Low-Dose/Low-Dose-Rate Radiation Induces Oxidative Damage in the Mouse Brain. Radiat. Res..

[10] Luo H., Wang C., Feng M., Zhao Y. (2014). Microgravity inhibits resting T cell immunity in an exposure time-dependent manner. Int. J. Med. Sci..

[11] Sanzari J.K., Romeroweaver A.L., James G., Krigsfeld G., Lin L., Diffenderfer E.S., Kennedy A.R. (2013). Leukocyte Activity Is Altered in a Ground Based Murine Model of Microgravity and Proton Radiation Exposure. PLoS ONE.

[12] Cazzaniga A., Maier J.A.M., Castiglioni S. (2016). Impact of simulated microgravity on human bone stem cells: New hints for space medicine. Biochem. Biophys. Res. Commun..

[13] Grimm D., Grosse J., Wehland M., Mann V., Reseland J.E., Sundaresan A., Corydon T.J. (2016). The impact of microgravity on bone in humans. Bone.

[14] Cappellesso R., Nicole L., Guido A., Pizzol D. (2015). Spaceflight osteoporosis: Current state and future perspective. Endocr. Regul..

[15] Sibonga J.D. (2013). Spaceflight-induced bone loss: Is there an osteoporosis risk?. Curr. Osteoporos. Rep..

[16] Lang T., Van Loon J., Bloomfield S., Vico L., Chopard A., Rittweger J., Kyparos A., Blottner D., Vuori I., Gerzer R. (2017). Towards human exploration of space: The THESEUS review series on muscle and bone research priorities. NPJ Microgravity.

[17] Markin A.A., Zhuravlëva O.A. (1993). Lipid peroxidation and antioxidant defense system in rats after a 14-day space flight in the “Space-2044” spacecraft. Aviakosm. Ekolog. Med..

[18] Markin A.A., Popova I.A., Vetrova E.G., Zhuravleva O.A., Balashov O.I. (1997). Lipid peroxidation and activity of diagnostically significant enzymes in cosmonauts after flights of various durations. Aviakosm. Ekolog. Med..

[19] Stein T.P., Leskiw M.J. (2000). Oxidant damage during and after spaceflight. Am. J. Physiol. Endocrinol. Metab..

[20] Balaban R.S., Nemoto S., Finkel T. (2005). Mitochondria, oxidants, and aging. Cell.

[21] Giorgio M., Migliaccio E., Orsini F., Paolucci D., Moroni M., Contursi C., Pelliccia G., Luzi L., Minucci S., Marcaccio M. (2005). Electron transfer between cytochrome c and p66Shc generates reactive oxygen species that trigger mitochondrial apoptosis. Cell.

[22] Quarrie J.K., Riabowol K.T. (2004). Murine models of life span extension. Sci. Aging Knowl. Environ..

[23] Finkel T., Holbrook N.J. (2000). Oxidants, oxidative stress and the biology of ageing. Nature.

[24] Glasauer A., Chandel N.S. (2013). Ros. Curr. Biol..

[25] Valko M., Leibfritz D., Moncol J., Cronin M.T., Mazur M., Telser J. (2007). Free radicals and antioxidants in normal physiological functions and human disease. Int. J. Biochem. Cell Biol..

[26] De Boer J., Andressoo J.O., de Wit J., Huijmans J., Beems R.B., van Steeg H., Weeda G., van der Horst G.T., van Leeuwen W., Themmen A.P. (2002). Premature aging in mice deficient in DNA repair and transcription. Science.

[27] Bai X.C., Lu D., Bai J., Zheng H., Ke Z.Y., Li X.M., Luo S.Q. (2004). Oxidative stress inhibits osteoblastic differentiation of bone cells by ERK and NF-kappaB. Biochem. Biophys. Res. Commun..

[28] Lean J.M., Davies J.T., Fuller K., Jagger C.J., Kirstein B., Partington G.A., Urry Z.L., Chambers T.J. (2003). A crucial role for thiol antioxidants in estrogen-deficiency bone loss. J. Clin. Investig..

[29] Almeida M., Han L., Martin-Millan M., Plotkin L.I., Stewart S.A., Roberson P.K., Kousteni S., O’Brien C.A., Bellido T., Parfitt A.M. (2007). Skeletal involution by age-associated oxidative stress and its acceleration by loss of sex steroids. J. Biol. Chem..

[30] Manolagas S.C. (2010). From estrogen-centric to aging and oxidative stress: A revised perspective of the pathogenesis of osteoporosis. Endocr. Rev..

[31] Almeida M., Han L., Martin-Millan M., O’Brien C.A., Manolagas S.C. (2007). Oxidative stress antagonizes Wnt signaling in osteoblast precursors by diverting beta-catenin from T cell factor- to forkhead box O-mediated transcription. J. Biol. Chem..

[32] Almeida M., Ambrogini E., Han L., Manolagas S.C., Jilka R.L. (2009). Increased lipid oxidation causes oxidative stress, increased peroxisome proliferator-activated receptor-gamma expression, and diminished pro-osteogenic Wnt signaling in the skeleton. J. Biol. Chem..

[33] Almeida M., Martin-Millan M., Ambrogini E., Bradsher R., Han L., Chen X.D., Roberson P.K., Weinstein R.S., O’Brien C.A., Jilka R.L. (2010). Estrogens attenuate oxidative stress and the differentiation and apoptosis of osteoblasts by DNA-binding-independent actions of the ERalpha. J. Bone. Miner. Res..

[34] Katoh M., Katoh M. (2004). Human FOX gene family (Review). Int. J. Oncol..

[35] Klotz L.O., Sanchez-Ramos C., Prieto-Arroyo I., Urbanek P., Steinbrenner H., Monsalve M. (2015). Redox regulation of FoxO transcription factors. Redox. Biol..

[36] Essers M.A., de Vries-Smits L.M., Barker N., Polderman P.E., Burgering B.M., Korswagen H.C. (2005). Functional interaction between beta-catenin and FOXO in oxidative stress signaling. Science.

[37] Staal F.J., Clevers H. (2000). Tcf/Lef transcription factors during T-cell development: Unique and overlapping functions. Hematol. J..

[38] Moon R.T., Bowerman B., Boutros M., Perrimon N. (2002). The promise and perils of Wnt signaling through beta-catenin. Science.

[39] Iyer S., Ambrogini E., Bartell S.M., Han L., Roberson P.K., de Cabo R., Jilka R.L., Weinstein R.S., O’Brien C.A., Manolagas S.C. (2013). FOXOs attenuate bone formation by suppressing Wnt signaling. J. Clin. Investig..

[40] Manolagas S.C., Almeida M. (2007). Gone with the Wnts: Beta-catenin, T-cell factor, forkhead box O, and oxidative stress in age-dependent diseases of bone, lipid, and glucose metabolism. Mol. Endocrinol..

[41] Rached M.T., Kode A., Xu L., Yoshikawa Y., Paik J.H., Depinho R.A., Kousteni S. (2010). FoxO1 is a positive regulator of bone formation by favoring protein synthesis and resistance to oxidative stress in osteoblasts. Cell Metab..

[42] Ambrogini E., Almeida M., Martin M. (2010). FoxO-Mediated Defense against Oxidative Stress in Osteoblasts Is Indispensable for Skeletal Homeostasis in Mice. Cell Metab..

[43] Bartell S.M., Kim H.N., Ambrogini E., Han L., Iyer S., Serra Ucer S., Rabinovitch P., Jilka R.L., Weinstein R.S., Zhao H. (2014). FoxO proteins restrain osteoclastogenesis and bone resorption by attenuating H_2_O_2_ accumulation. Nat. Commun..

[44] Zhu H., Zhang L., Itoh K., Yamamoto M., Ross D., Trush M.A., Zweier J.L., Li Y. (2006). Nrf2 controls bone marrow stromal cell susceptibility to oxidative and electrophilic stress. Free. Radic. Biol. Med..

[45] Gan X., Huang S., Yu Q., Yu H., Yan S.S. (2015). Blockade of Drp1 rescues oxidative stress-induced osteoblast dysfunction. Biochem. Biophys. Res. Commun..

[46] Yang Y.H., Li B., Zheng X.F., Chen J.W., Chen K., Jiang S.D., Jiang L.S. (2014). Oxidative damage to osteoblasts can be alleviated by early autophagy through the endoplasmic reticulum stress pathway--implications for the treatment of osteoporosis. Free Radic. Biol. Med..

[47] Almeida M., O’Brien C.A. (2013). Basic biology of skeletal aging: Role of stress response pathways. J. Gerontol. A Biol. Sci. Med. Sci..

[48] Smith S.M., Heer M. (2002). Calcium and bone metabolism during space flight. Nutrition.

[49] Smith S.M., Heer M.A., Shackelford L.C., Sibonga J.D., Ploutz-Snyder L., Zwart S.R. (2012). Benefits for bone from resistance exercise and nutrition in long-duration spaceflight: Evidence from biochemistry and densitometry. J. Bone Miner. Res..

[50] Smith S.M., Wastney M.E., O’Brien K.O., Morukov B.V., Larina I.M., Abrams S.A., Davis-Street J.E., Oganov V., Shackelford L.C. (2005). Bone Markers, Calcium Metabolism, and Calcium Kinetics During Extended-Duration Space Flight on the Mir Space Station. J. Bone Miner. Res. Off. J. Am. Soc. Bone Miner. Res..

[51] Morgan J.L., Zwart S.R., Heer M., Ploutz-Snyder R., Ericson K., Smith S.M. (2012). Bone metabolism and nutritional status during 30-day head-down-tilt bed rest. J. Appl. Physiol. (1985).

[52] LeBlanc A., Schneider V., Shackelford L., West S., Oganov V., Bakulin A., Voronin L. (2000). Bone mineral and lean tissue loss after long duration space flight. J. Musculoskelet. Neuronal Interact..

[53] Zwart S.R., Oliver S.A., Fesperman J.V., Kala G., Krauhs J., Ericson K., Smith S.M. (2009). Nutritional status assessment before, during, and after long-duration head-down bed rest. Aviat. Space Environ. Med..

[54] Smith S.M., Zwart S.R., Block G., Rice B.L., Davis-Street J.E. (2005). The nutritional status of astronauts is altered after long-term space flight aboard the International Space Station. J. Nutr..

[55] Zwart S.R., Morgan J.L., Smith S.M. (2013). Iron status and its relations with oxidative damage and bone loss during long-duration space flight on the International Space Station. Am. J. Clin. Nutr..

[56] Rizzo A.M., Corsetto P.A., Montorfano G., Milani S., Zava S., Tavella S., Cancedda R., Berra B. (2012). Effects of long-term space flight on erythrocytes and oxidative stress of rodents. PLoS ONE.

[57] Lawler J.M., Song W., Demaree S.R. (2003). Hindlimb unloading increases oxidative stress and disrupts antioxidant capacity in skeletal muscle. Free Radic. Biol. Med..

[58] Chowdhury P., Soulsby M., Kim K. (2007). L-carnitine influence on oxidative stress induced by hind limb unloading in adult rats. Aviat. Space Environ. Med..

[59] Xin M., Yang Y., Zhang D., Wang J., Chen S., Zhou D. (2015). Attenuation of hind-limb suspension-induced bone loss by curcumin is associated with reduced oxidative stress and increased vitamin D receptor expression. Osteoporos. Int..

[60] Sun Y., Shuang F., Chen D.M., Zhou R.B. (2013). Treatment of hydrogen molecule abates oxidative stress and alleviates bone loss induced by modeled microgravity in rats. Osteoporos. Int..

[61] Takahashi K., Okumura H., Guo R., Naruse K. (2017). Effect of Oxidative Stress on Cardiovascular System in Response to Gravity. Int. J. Mol. Sci..

[62] Smith S.M., Davis-Street J.E., Fesperman J.V., Smith M.D., Rice B.L., Zwart S.R. (2004). Nutritional status changes in humans during a 14-day saturation dive: The NASA Extreme Environment Mission Operations V project. J. Nutr..

[63] Hollander J., Gore M., Fiebig R., Mazzeo R., Ohishi S., Ohno H., Ji L. (1998). Spaceflight downregulates antioxidant defense systems in rat liver. Free Radic. Biol. Med..

[64] Nojiri H., Saita Y., Morikawa D., Kobayashi K., Tsuda C., Miyazaki T., Saito M., Marumo K., Yonezawa I., Kaneko K., Shirasawa T., Shimizu T. (2011). Cytoplasmic superoxide causes bone fragility owing to low-turnover osteoporosis and impaired collagen cross-linking. J. Bone Miner. Res..

[65] Morikawa D., Nojiri H., Saita Y., Kobayashi K., Watanabe K., Ozawa Y., Koike M., Asou Y., Takaku T., Kaneko K., Shimizu T. (2013). Cytoplasmic reactive oxygen species and SOD1 regulate bone mass during mechanical unloading. J. Bone Miner. Res..

[66] Beck M., Moreels M., Quintens R., Abou-El-Ardat K., El-Saghire H., Tabury K., Michaux A., Janssen A., Neefs M., Van Oostveldt P. (2014). Chronic exposure to simulated space conditions predominantly affects cytoskeleton remodeling and oxidative stress response in mouse fetal fibroblasts. Int. J. Mol. Med..

[67] Suman S., Rodriguez O.C., Winters T.A., Fornace A.J., Albanese C., Datta K. (2013). Therapeutic and space radiation exposure of mouse brain causes impaired DNA repair response and premature senescence by chronic oxidant production. Aging.

[68] Christofidou-Solomidou M., Pietrofesa R.A., Arguiri E., Schweitzer K.S., Berdyshev E.V., McCarthy M., Corbitt A., Alwood J.S., Yu Y., Globus R.K. (2015). Space radiation-associated lung injury in a murine model. Am. J. Physiol. Lung Cell. Mol. Physiol..

[69] Guan J., Stewart J., Ware J.H., Zhou Z., Donahue J.J., Kennedy A.R. (2006). Effects of dietary supplements on the space radiation-induced reduction in total antioxidant status in CBA mice. Radiat. Res..

[70] Kook S.H., Kim K.A., Ji H., Lee D., Lee J.C. (2015). Irradiation inhibits the maturation and mineralization of osteoblasts via the activation of Nrf2/HO-1 pathway. Mol. Cell Biochem..

[71] Kondo H., Limoli C., Searby N.D., Almeida E.A., Loftus D.J., Vercoutere W., Morey-Holton E., Giedzinski E., Mojarrab R., Hilton D. (2007). Shared oxidative pathways in response to gravity-dependent loading and gamma-irradiation of bone marrow-derived skeletal cell progenitors. Radiatsionnaia Biol. Radioecol..

[72] Kondo H., Yumoto K., Alwood J.S., Mojarrab R., Wang A., Almeida E.A., Searby N.D., Limoli C.L., Globus R.K. (2010). Oxidative stress and gamma radiation-induced cancellous bone loss with musculoskeletal disuse. J. Appl. Physiol. (1985).

[73] Maillet A., Beaufrere B., Di N.P., Elia M., Pichard C. (2001). Weightlessness as an accelerated model of nutritional disturbances. Curr. Opin. Clin. Nutr. Metab. Care.

[74] Bergouignan A., Stein T.P., Habold C., Coxam V., O’Gorman D., Blanc S. (2016). Towards human exploration of space: The THESEUS review series on nutrition and metabolism research priorities. NPJ Microgravity.

[75] Yang Y., Su Y., Wang D., Chen Y., Wu T., Li G., Sun X., Cui L. (2013). Tanshinol attenuates the deleterious effects of oxidative stress on osteoblastic differentiation via Wnt/FoxO3a signaling. Oxid. Med. Cell. Longev..

[76] Zeng X., Tian J., Cai K., Wu X., Wang Y., Zheng Y., Su Y., Cui L. (2014). Promoting osteoblast differentiation by the flavanes from Huangshan Maofeng tea is linked to a reduction of oxidative stress. Phytomed. Int. J. Phytother. Phytopharmacol..

[77] Vester H., Holzer N., Neumaier M., Lilianna S., Nussler A.K., Seeliger C. (2014). Green Tea Extract (GTE) improves differentiation in human osteoblasts during oxidative stress. J. Inflamm..

